# Effect of N-terminal pro B-type natriuretic peptide levels on the efficacy and safety of esaxerenone vs trichlormethiazide for the treatment of Japanese patients with uncontrolled essential hypertension: a subanalysis of the EXCITE-HT study

**DOI:** 10.1038/s41440-025-02412-8

**Published:** 2025-11-07

**Authors:** Kazuomi Kario, Mitsuru Ohishi, Tomohiro Katsuya, Tatsuo Shimosawa, Kazuhito Shiosakai, Taketoshi Furugori, Takashi Taguchi

**Affiliations:** 1 Division of Cardiovascular Medicine, Department of Medicine, Jichi Medical University School of Medicine, Shimotsuke, Tochigi Japan; 2 Department of Cardiovascular Medicine and Hypertension, Graduate School of Medical and Dental Sciences, Kagoshima University, Kagoshima, Kagoshima Japan; 3 Katsuya Clinic, Amagasaki, Hyogo Japan; 4 Department of Clinical Laboratory, School of Medicine, International University of Health and Welfare, Narita, Chiba Japan; 5 Data Intelligence Department, Daiichi Sankyo Co. Ltd., Shinagawa-ku, Tokyo Japan; 6 Primary Medical Science Department, Medical Affairs Division, Daiichi Sankyo Co. Ltd., Chuo-ku, Tokyo Japan

**Keywords:** Esaxerenone, Japan, Morning home blood pressure, NT-proBNP, Trichlormethiazide

## Abstract

It is currently unknown whether baseline N-terminal pro B-type natriuretic peptide (NT-proBNP) levels, a marker of pressure-related cardiac stress, affect the efficacy and safety of esaxerenone vs trichlormethiazide. This exploratory subanalysis of the EXCITE-HT study aimed to compare the antihypertensive efficacy and safety of esaxerenone vs trichlormethiazide in Japanese patients with uncontrolled essential hypertension stratified by baseline NT-proBNP. EXCITE-HT was a randomized, open-label, parallel-group study. In this subanalysis, patients were divided into low and high NT-proBNP subgroups based on their baseline levels (<125 pg/mL and ≥125 pg/mL, respectively). The low NT-proBNP subgroup included 188 and 212 patients and the high NT-proBNP subgroup included 49 and 41 patients in the esaxerenone and trichlormethiazide groups, respectively. A significant decrease in morning home systolic/diastolic blood pressure (SBP/DBP; the primary endpoint) was observed from baseline to the end of treatment in both NT-proBNP subgroups. In the low NT-proBNP subgroup, esaxerenone was superior to trichlormethiazide in lowering SBP and non-inferior in lowering DBP. Non-inferiority could not be confirmed in the high NT-proBNP subgroup. The geometric mean urinary albumin-to-creatinine ratio significantly decreased from baseline to Week 12 in all subgroups (all *P* < 0.001 vs baseline). No cases of serum potassium level ≥6.0 mEq/L were reported. These findings suggest that esaxerenone is efficacious across a wide range of baseline NT-proBNP levels, while also supporting its favorable safety profile.

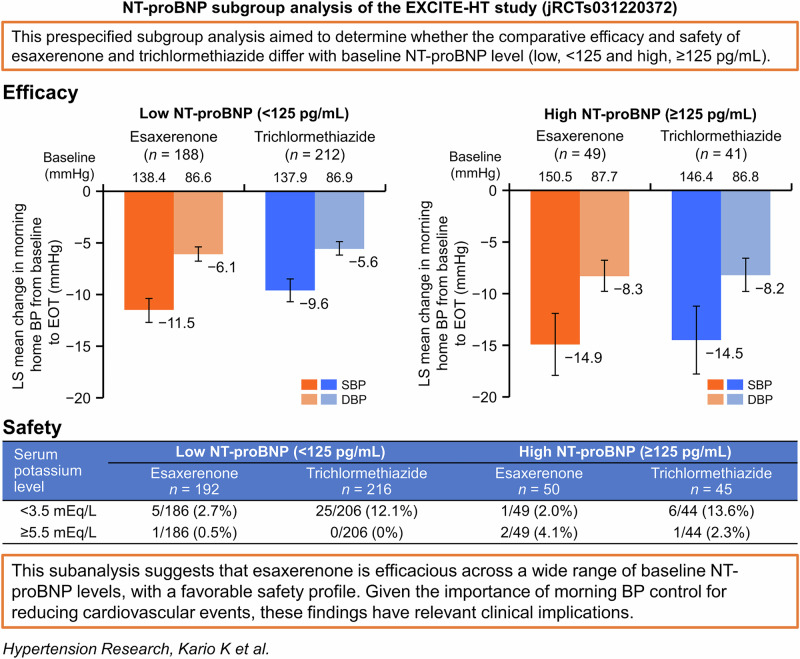

## Introduction

Heart failure (HF) represents a major clinical concern for patients with hypertension, as the presence of diastolic or systolic dysfunction increases both morbidity and mortality [1]. N-terminal pro B-type natriuretic peptide (NT-proBNP) is widely recognized as a surrogate marker for HF severity and cardiac stress [2, 3], based on its close association with left ventricular dysfunction and its established diagnostic and prognostic utility [4, 5]. Patients with heart disease—especially HF—are prone to hypokalemia, and diuretics have a class effect of decreasing potassium levels. These factors highlight the essential balance between effective blood pressure (BP) control and electrolyte management in hypertensive patients with HF (elevated NT-proBNP levels), especially when treated with diuretics.

For hypertensive patients with HF, the 2019 Japanese Society of Hypertension (JSH) guidelines recommend drugs that have been shown to be clinically effective in the treatment of HF, including renin–angiotensin system inhibitors, beta-blockers, and mineralocorticoid receptor blockers (MRBs) [6]. However, despite the value of NT-proBNP as a marker of HF and organ damage, there is insufficient evidence on the optimal use of this marker in actual clinical practice to guide antihypertensive treatment decisions. In particular, patients whose NT-proBNP levels are elevated may be more likely to have impaired left ventricular systolic or diastolic function, suggesting that MRBs and other agents could be indicated. However, it remains unclear whether the effectiveness and safety of antihypertensive medications differ based on baseline NT-proBNP levels.

Esaxerenone is a next-generation non-steroidal MRB with higher selectivity and potency, longer half-life, and more favorable bioavailability compared with other MRBs [7, 8]. The ESES-LVH study demonstrated that esaxerenone elicited favorable antihypertensive and cardioprotective effects, including a reduction in left ventricular mass index, in patients with uncontrolled hypertension and left ventricular hypertrophy [9]. Additionally, ESES-LVH and ENaK (both single-arm studies) [9, 10], demonstrated that esaxerenone significantly reduced NT-proBNP levels regardless of the concomitant antihypertensive medication (angiotensin receptor blocker [ARB] or calcium channel blocker [CCB]). The EXCITE-HT study compared esaxerenone and trichlormethiazide as add-on therapy in patients with uncontrolled essential hypertension receiving an ARB or CCB, showing that esaxerenone was non-inferior in lowering morning home BP [11–14]. Although the primary EXCITE-HT study was designed primarily to evaluate the non-inferiority of esaxerenone vs trichlormethiazide in patients with uncontrolled essential hypertension, many of these patients reflected a real-world population in which HF risk is not uncommon.

Given the established role of NT-proBNP as a practical surrogate for HF status in routine clinical practice, this subanalysis selected it as a key stratification variable to investigate whether baseline NT-proBNP levels (<125 pg/mL or ≥125 pg/mL) influence the comparative efficacy and safety of esaxerenone and trichlormethiazide.

## Methods

### Study design

The EXCITE-HT study was a randomized, open-label, parallel-group study conducted from December 2022 to September 2023 at 54 study sites [11, 12]. In this subanalysis, patients were allocated to two subgroups based on baseline NT-proBNP levels (low NT-proBNP subgroup: <125 pg/mL and high NT-proBNP subgroup: ≥125 pg/mL). This threshold was chosen according to established clinical guidelines, both in Japan and internationally [15, 16]. NT-proBNP levels were measured at each medical institution participating in the study rather than at a central laboratory. The main study was approved by the Certified Review Board of Hattori Clinic (CRB3180027) and was registered at the Japan Registry of Clinical Trials under the identifier jRCTs031220372. The EXCITE-HT study was conducted in accordance with the principles of the Declaration of Helsinki and the Clinical Trials Act in Japan. All patients provided written informed consent prior to enrollment.

### Patients

Patients ≥20 years of age who had previously received treatment with either one ARB or one CCB at the same dose for ≥4 weeks before registration and with a mean morning home systolic BP (SBP) and/or diastolic BP (DBP) ≥ 125 mmHg and/or ≥75 mmHg, respectively, were included in the EXCITE-HT study [11, 12]. Patients ≥75 years of age with cerebrovascular disease or proteinuria-negative chronic kidney disease were only eligible for inclusion if their mean morning home SBP and/or DBP was ≥135 mmHg and/or ≥85 mmHg, respectively. This subanalysis included patients from the EXCITE-HT study with available NT-proBNP measurements.

### Study interventions

Esaxerenone was administered for 12 weeks, in accordance with the Japanese package insert [17], at a starting dose of 2.5 mg/day. In patients with creatinine-based estimated glomerular filtration rate (eGFR_creat_) of 30–59 mL/min/1.73 m^2^ or in those with diabetes mellitus and albuminuria at baseline, the starting dose was 1.25 mg/day. After 4 or 8 weeks of treatment, the dose could be gradually increased to a maximum of 5 mg/day, based on BP measurement, serum potassium level, and the clinical judgement of the attending physician.

Trichlormethiazide was prescribed based on the discretion of the attending physician, in accordance with the Japanese package insert and 2019 JSH guidelines [6, 18], which recommend a starting dose of ≤1 mg/day. Depending on the patient’s condition and the clinical judgement of the attending physician, the dose could be increased after 4 or 8 weeks of treatment.

Basal antihypertensive drugs (ARBs or CCBs) were administered at a fixed dose throughout the treatment period until the end of treatment (EOT), and the use of other antihypertensive drugs was prohibited.

### Study endpoints

For the present subanalysis, the primary endpoint (change in morning home SBP/DBP from baseline to EOT), secondary endpoints (change in bedtime home and office SBP/DBP from baseline to EOT and change in urinary albumin-to-creatinine ratio [UACR] and serum NT-proBNP levels from baseline to Week 12), and safety endpoints of the primary EXCITE-HT study were evaluated in two patient subgroups based on their baseline NT-proBNP levels (low and high NT-proBNP subgroups). The safety endpoints were treatment-emergent adverse events (TEAEs) coded using the Medical Dictionary for Regulatory Activities (MedDRA)/Japanese, version 25.1; the time-course changes and change from baseline in eGFR_creat_ and serum potassium throughout the study period; the proportion of patients with serum potassium levels ≤3.5 mEq/L, ≥5.5 mEq/L, or ≥6.0 mEq/L; and the proportion of patients with uric acid (UA) level >7.0 mg/dL.

### Sample size and statistical analyses

The target sample size was determined for the primary study; it was not specifically determined for this subanalysis. This was an exploratory subanalysis that examined the non-inferiority of esaxerenone vs trichlormethiazide using the same statistical methods as the primary analysis [11, 12]. In this subanalysis, the non-inferiority criteria established in the primary analysis were used to evaluate non-inferiority, and the non-inferiority margins from the primary analysis (3.9 mmHg for SBP and 2.1 mmHg for DBP) were referenced when interpreting the subanalysis results. Esaxerenone was considered superior to trichlormethiazide in its BP-lowering effect if the upper limit of the two-sided 95% CI was <0 mmHg. The analysis for efficacy was conducted using the full analysis set (FAS) and that for safety was conducted using the safety analysis set. These definitions have been previously reported [11]. All statistical analyses were conducted with a two-sided significance level of 5%, unless otherwise stated. The statistical software used was SAS version 9.4 or later (SAS Institute Inc., Cary, NC, USA).

## Results

### Patients

In the primary EXCITE-HT study, 600 patients were eligible and randomly assigned to the esaxerenone and trichlormethiazide groups (295 and 290 patients, respectively, in the FAS; 302 and 298 patients, respectively, in the safety analysis set) [12]. In the FAS for this subanalysis, the low NT-proBNP subgroup included 188 and 212 patients in the esaxerenone and trichlormethiazide groups, respectively, and the high NT-proBNP subgroup included 49 and 41 patients in the esaxerenone and trichlormethiazide groups, respectively.

Baseline patient demographic and clinical characteristics in the NT-proBNP subgroups are shown in Table 1. Patient characteristics were well balanced between the esaxerenone and trichlormethiazide groups in each NT-proBNP subgroup. Compared with the low NT-proBNP subgroup, the high NT-proBNP subgroup had a higher mean age, greater proportion of patients aged ≥65 years, and higher mean UACR, along with a lower mean body mass index (BMI), smaller proportion of patients with BMI ≥ 25 kg/m^2^, and lower mean eGFR_creat_. The proportion of patients with HF as a complication was higher in the high NT-proBNP subgroup (esaxerenone: 24.5% [12/49]; trichlormethiazide: 22.0% [9/41]) than in the low NT-proBNP subgroup (esaxerenone: 5.3% [10/188]; trichlormethiazide: 3.3% [7/212]). The high NT-proBNP subgroup had longer mean disease duration of hypertension and higher mean morning home, bedtime home, and office BP compared with the low NT-proBNP subgroup (esaxerenone and trichlormethiazide: duration of hypertension, 5.61 and 7.33 years vs 5.01 and 5.15 years; mean morning home BP, 150.5/87.7 and 146.4/86.8 mmHg vs 138.4/86.6 and 137.9/86.9 mmHg; mean bedtime home BP, 140.6/80.3 and 135.4/78.4 mmHg vs 133.8/81.7 and 134.5/82.5 mmHg; mean office BP, 155.1/82.8 and 145.7/80.5 mmHg vs 142.5/83.2 and 142.6/84.5 mmHg, respectively). No notable differences were observed in the distribution of esaxerenone or trichlormethiazide doses between the NT-proBNP subgroups; however, no confirmatory statistical tests were performed.

**Table 1:** Baseline patient demographic and clinical characteristics in the NT-proBNP subgroups (full analysis set)

Characteristics	Low NT-proBNP (<125 pg/mL)		High NT-proBNP (≥125 pg/mL)	
	Esaxerenone *n* = 188	Trichlormethiazide *n* = 212	Esaxerenone *n* = 49	Trichlormethiazide *n* = 41
Sex, male	93 (49.5)	113 (53.3)	21 (42.9)	20 (48.8)
Age, years	63.6 ± 11.2	62.8 ± 11.9	74.8 ± 8.9	73.7 ± 9.0
≥65	91 (48.4)	102 (48.1)	42 (85.7)	35 (85.4)
Body mass index, kg/m^2^	25.94 ± 4.30	25.77 ± 4.13	23.02 ± 3.26	23.87 ± 4.08
≥25	98 (52.1)	122 (57.5)	14 (28.6)	12 (29.3)
Morning home SBP, mmHg	138.4 ± 13.4	137.9 ± 13.0	150.5 ± 17.9	146.4 ± 13.2
Morning home DBP, mmHg	86.6 ± 9.4	86.9 ± 8.9	87.7 ± 11.5	86.8 ± 11.5
Bedtime home SBP, mmHg	133.8 ± 13.8 *n* = 179	134.5 ± 13.4 *n* = 206	140.6 ± 22.0 *n* = 44	135.4 ± 18.3 *n* = 40
Bedtime home DBP, mmHg	81.7 ± 9.9 *n* = 179	82.5 ± 10.1 *n* = 206	80.3 ± 12.9 *n* = 44	78.4 ± 13.4 *n* = 40
Office SBP, mmHg	142.5 ± 14.7	142.6 ± 14.8	155.1 ± 19.4	145.7 ± 17.1
Office DBP, mmHg	83.2 ± 11.1	84.5 ± 12.4	82.8 ± 13.5	80.5 ± 12.3
NT-proBNP, pg/mL	43.44 ± 31.77	46.01 ± 33.76	362.94 ± 617.81	279.46 ± 289.85
Median (range)	38.00 (0.50, 121.00)	36.35 (0.50, 124.00)	185.00 (126.00, 4229.00)	183.00 (129.00, 1763.00)
<55	125 (66.5)	140 (66.0)	0	0
55 to <125	63 (33.5)	72 (34.0)	0	0
≥125	0	0	49 (100.0)	41 (100.0)
UACR, mg/gCr	53.88 ± 120.10	63.57 ± 146.67	421.90 ± 1099.92	300.10 ± 1024.86
<30	127 (67.6)	141 (66.5)	23 (46.9)	22 (53.7)
30 to <300	55 (29.3)	59 (27.8)	16 (32.7)	13 (31.7)
≥300	6 (3.2)	12 (5.7)	10 (20.4)	6 (14.6)
Serum potassium, mEq/L	4.18 ± 0.36 *n* = 181	4.21 ± 0.32 *n* = 201	4.29 ± 0.34 *n* = 47	4.26 ± 0.32 *n* = 40
Uric acid, mg/dL	5.37 ± 1.31 *n* = 188	5.43 ± 1.21 *n* = 212	5.34 ± 1.20 *n* = 49	5.48 ± 1.30 *n* = 40
eGFR_creat_, mL/min/1.73 m^2^	73.37 ± 15.85 *n* = 188	73.57 ± 15.63 *n* = 209	63.78 ± 15.00 *n* = 49	64.66 ± 21.78 *n* = 41
Duration of hypertension, years	5.01 ± 4.75 *n* = 133	5.15 ± 4.83 *n* = 148	5.61 ± 4.94 *n* = 35	7.33 ± 5.91 *n* = 24
Complications				
T2DM	77 (41.0)	79 (37.3)	21 (42.9)	16 (39.0)
Dyslipidemia	122 (64.9)	124 (58.5)	25 (51.0)	27 (65.9)
Hyperuricemia	31 (16.5)	34 (16.0)	4 (8.2)	5 (12.2)
Heart failure	10 (5.3)	7 (3.3)	12 (24.5)	9 (22.0)
Esaxerenone dose at baseline (initial dose), mg				
1.25	67 (35.6)	–	29 (59.2)	–
2.5	121 (64.4)	–	20 (40.8)	–
Esaxerenone dose at EOT (last dose), mg				
1.25	40 (21.3)	–	14 (28.6)	–
2.5	115 (61.2)	–	24 (49.0)	–
5	33 (17.6)	–	11 (22.4)	–
Trichlormethiazide dose at baseline (initial dose), mg				
0.25	–	4 (1.9)	–	0
0.5	–	15 (7.1)	–	2 (4.9)
1	–	189 (89.2)	–	37 (90.2)
2	–	4 (1.9)	–	2 (4.9)
Trichlormethiazide dose at EOT (last dose), mg				
0.25	–	2 (0.9)	–	0
0.5	–	15 (7.1)	–	3 (7.3)
1	–	182 (85.8)	–	34 (82.9)
>1 to ≤2	–	11 (5.2)	–	4 (9.8)
≥3	–	2 (0.9)	–	0
Basal antihypertensive agent				
ARB	70 (37.2)	77 (36.3)	26 (53.1)	23 (56.1)
CCB	118 (62.8)	135 (63.7)	23 (46.9)	18 (43.9)

Data are *n* (%) or mean ± standard deviation, unless otherwise stated

*ARB angiotensin receptor blocker, CCB calcium channel blocker, DBP* diastolic blood pressure, *eGFR*_*creat*_ creatinine-based estimated glomerular filtration rate, *EOT* end of treatment, *NT-proBNP* N-terminal pro B-type natriuretic peptide, *SBP* systolic blood pressure, *T2DM* type 2 diabetes mellitus, *UACR* urinary albumin-to-creatinine ratio

### BP-lowering effects

**Fig. 1:**
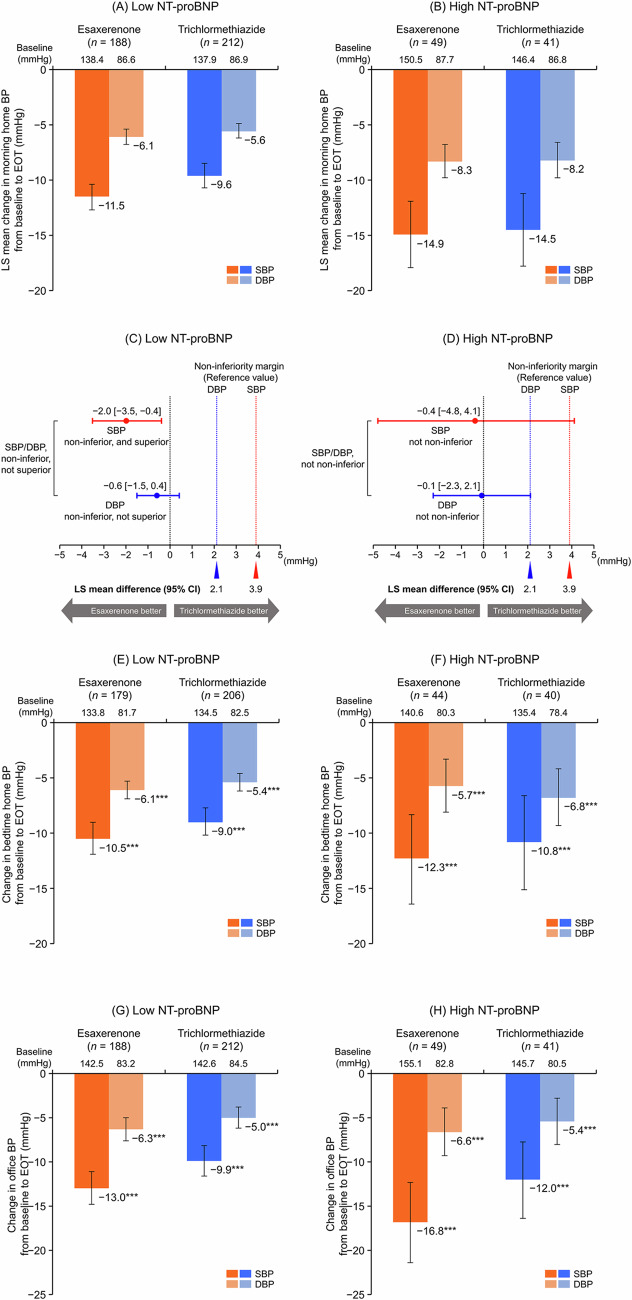
Changes from baseline to EOT in **A–D** morning home, **E**, **F** bedtime home, and **G**, **H** office BP (full analysis set). **A**, **C**, **E**, **G** Low NT-proBNP subgroup (<125 pg/mL); **B**, **D**, **F**, **H** high NT-proBNP subgroup (≥ 125 pg/mL). For panels **C** and **D**, the red dotted line (3.9 mmHg) and blue dotted line (2.1 mmHg) indicate the non-inferiority criteria. Data are LS mean (95% CI) for panels (**A–D**). Data are arithmetic mean (95% CI) for panels (**E–H**). ***P < 0.001 versus baseline, paired *t*-test. BP blood pressure, CI confidence interval, DBP diastolic blood pressure, EOT end of treatment, LS least squares, NT-proBNP N-terminal pro B-type natriuretic peptide, SBP systolic blood pressure

A significant decrease in morning home SBP/DBP was observed from baseline to EOT in both NT-proBNP subgroups (Supplementary Table 1). The least squares (LS) mean changes in morning home SBP/DBP from baseline to EOT were −11.5 (95% CI, −12.7, −10.4)/−6.1 (−6.8, −5.4) and −9.6 (−10.7, −8.5)/−5.6 (−6.2, −4.9) mmHg in the esaxerenone and trichlormethiazide groups, respectively, within the low NT-proBNP subgroup (Fig. 1A) and −14.9 (−17.9, −11.9)/−8.3 (−9.8, −6.8) and −14.5 (−17.8, −11.2)/−8.2 (−9.8, −6.6) mmHg, respectively, within the high NT-proBNP subgroup (Fig. 1B). The between-group difference in LS mean change was −2.0 (95% CI, −3.5, −0.4)/−0.6 (−1.5, 0.4) mmHg in the low NT-proBNP subgroup (Fig. 1C) and −0.4 (95% CI, −4.8, 4.1)/−0.1 (− 2.3, 2.1) mmHg in the high NT-proBNP subgroup (Fig. 1D). Significant reductions were also shown in bedtime home and office SBP/DBP in both NT-proBNP subgroups (all *P* < 0.001) (Fig. 1E–H and Supplementary Table 1). Although no statistical tests were performed, changes from baseline in morning home, bedtime home, and office BP were numerically greater in the high NT-proBNP subgroup than the low NT-proBNP subgroup.

### UACR and NT-proBNP

**Fig. 2:**
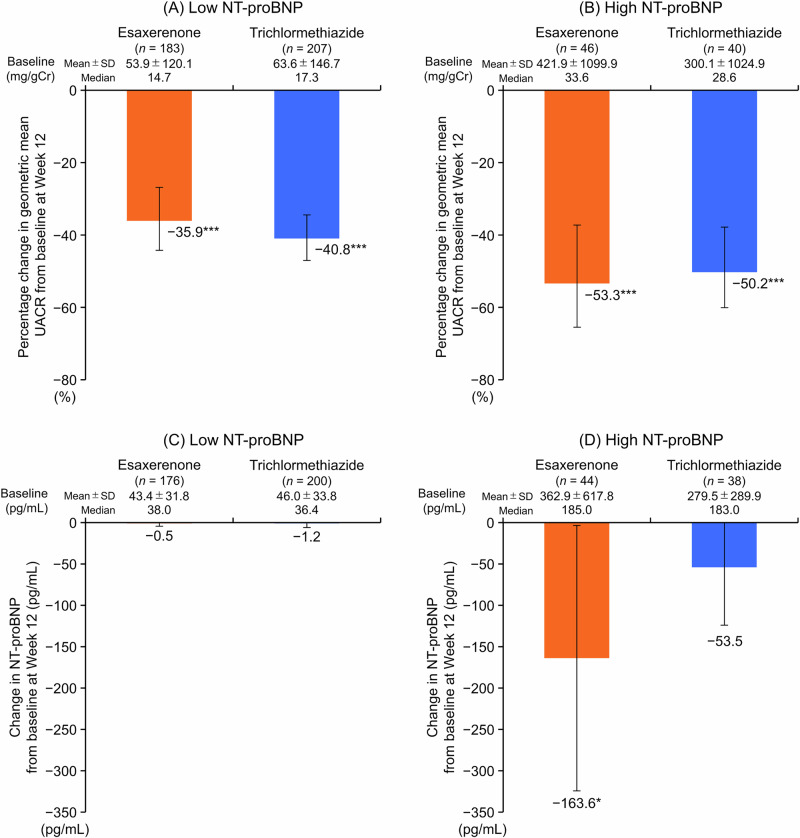
Percentage change in geometric mean UACR (**A**, **B**) and change from baseline in NT-proBNP (**C**, **D**) at Week 12 (full analysis set). **A**, **C** Low NT-proBNP subgroup (<125 pg/mL); **B**, **D** high NT-proBNP subgroup (≥125 pg/mL). Data are mean (95% CI). *P < 0.05 versus baseline, paired *t*-test. ***P < 0.001 versus baseline, paired *t*-test. CI confidence interval, NT-proBNP N-terminal pro B-type natriuretic peptide, SD standard deviation, UACR urinary albumin-to-creatinine ratio

The geometric mean UACR significantly decreased from baseline to Week 12 in all subgroups (low NT-proBNP subgroup: −35.9% for esaxerenone, −40.8% for trichlormethiazide; high NT-proBNP subgroup: −53.3% for esaxerenone, −50.2% for trichlormethiazide; all *P* < 0.001 vs baseline) (Fig. 2A, B, and Supplementary Table 2). The change from baseline in the geometric mean UACR was numerically greater in the high NT-proBNP subgroup than the low NT-proBNP subgroup.

Changes in NT-proBNP levels from baseline to Week 12 were similar between the esaxerenone and trichlormethiazide groups in the low NT-proBNP subgroup (Fig. 2C, Supplementary Table 2). In the high NT-proBNP subgroup, NT-proBNP levels were significantly reduced from baseline at Week 12 in the esaxerenone group, whereas no statistically significant reduction was observed in the trichlormethiazide group (Fig. 2D, Supplementary Table 2).

### Safety

TEAEs in the NT-proBNP subgroups are shown in Table 2. In the low NT-proBNP subgroup, TEAEs occurred in 71 (37.0%) and 88 (40.7%) patients in the esaxerenone and trichlormethiazide groups, respectively; in the high NT-proBNP subgroup, TEAEs occurred in 14 (28.0%) and 18 (40.0%) patients, respectively. Regarding TEAEs related to serum potassium, in the low NT-proBNP subgroup, blood potassium increased and blood potassium decreased (MedDRA preferred terms) occurred in three (1.6%) and zero patients in the esaxerenone group and zero and two (0.9%) patients in the trichlormethiazide group, respectively. In the high NT-proBNP subgroup, these events occurred in two (4.0%) and zero patients in the esaxerenone group and zero and one (2.2%) patient in the trichlormethiazide group, respectively. No cases of hyperkalemia were reported.

**Table 2:** TEAEs in the NT-proBNP subgroups (safety analysis set)

Type of TEAE	Low NT-proBNP (<125 pg/mL)		High NT-proBNP (≥125 pg/mL)	
	Esaxerenone *n* = 192	Trichlormethiazide *n* = 216	Esaxerenone *n* = 50	Trichlormethiazide *n* = 45
Any TEAEs	71 (37.0)	88 (40.7)	14 (28.0)	18 (40.0)
Frequent TEAEs (occurring in ≥2 patients in either treatment group)				
Nasopharyngitis	11 (5.7)	8 (3.7)	0	1 (2.2)
Dizziness	4 (2.1)	5 (2.3)	1 (2.0)	2 (4.4)
Hyperuricemia	2 (1.0)	9 (4.2)	0	1 (2.2)
Bronchitis	6 (3.1)	3 (1.4)	0	2 (4.4)
Back pain	3 (1.6)	3 (1.4)	0	0
COVID-19	3 (1.6)	3 (1.4)	0	0
Diarrhea	3 (1.6)	2 (0.9)	1 (2.0)	0
Headache	4 (2.1)	2 (0.9)	0	0
Abdominal pain upper	1 (0.5)	4 (1.9)	0	0
Blood potassium increased	3 (1.6)	0	2 (4.0)	0
Blood uric acid increased	0	4 (1.9)	1 (2.0)	0
Arthralgia	2 (1.0)	3 (1.4)	0	0
Malaise	3 (1.6)	2 (0.9)	0	0
Myalgia	2 (1.0)	1 (0.5)	0	2 (4.4)
Rhinitis allergic	3 (1.6)	2 (0.9)	0	0
Conjunctivitis allergic	3 (1.6)	1 (0.5)	0	0
Contusion	2 (1.0)	2 (0.9)	0	0
Dermatitis	2 (1.0)	2 (0.9)	0	0
Eczema	3 (1.6)	1 (0.5)	0	0
Gastroenteritis	2 (1.0)	1 (0.5)	1 (2.0)	0
Glomerular filtration rate decreased	1 (0.5)	3 (1.4)	0	0
Hypokalemia	0	3 (1.4)	0	1 (2.2)
Pharyngitis	2 (1.0)	1 (0.5)	1 (2.0)	0
Pyrexia	2 (1.0)	2 (0.9)	0	0
Upper respiratory tract inflammation	3 (1.6)	0	1 (2.0)	0
Urine albumin/creatinine ratio increased	3 (1.6)	1 (0.5)	0	0
Blood potassium decreased	0	2 (0.9)	0	1 (2.2)
Blood sodium decreased	0	2 (0.9)	0	1 (2.2)
Type 2 diabetes mellitus	0	3 (1.4)	0	0
Decreased appetite	0	2 (0.9)	0	0
Dermatitis contact	0	2 (0.9)	0	0
Influenza	0	2 (0.9)	0	0

Data are *n* (%). TEAEs were coded by Preferred Term using the Medical Dictionary for Regulatory Activities/Japanese, version 25.1

*NT-proBNP* N-terminal pro B-type natriuretic peptide, *TEAE* treatment-emergent adverse event

### eGFR_creat_, serum potassium, and UA levels

**Fig. 3:**
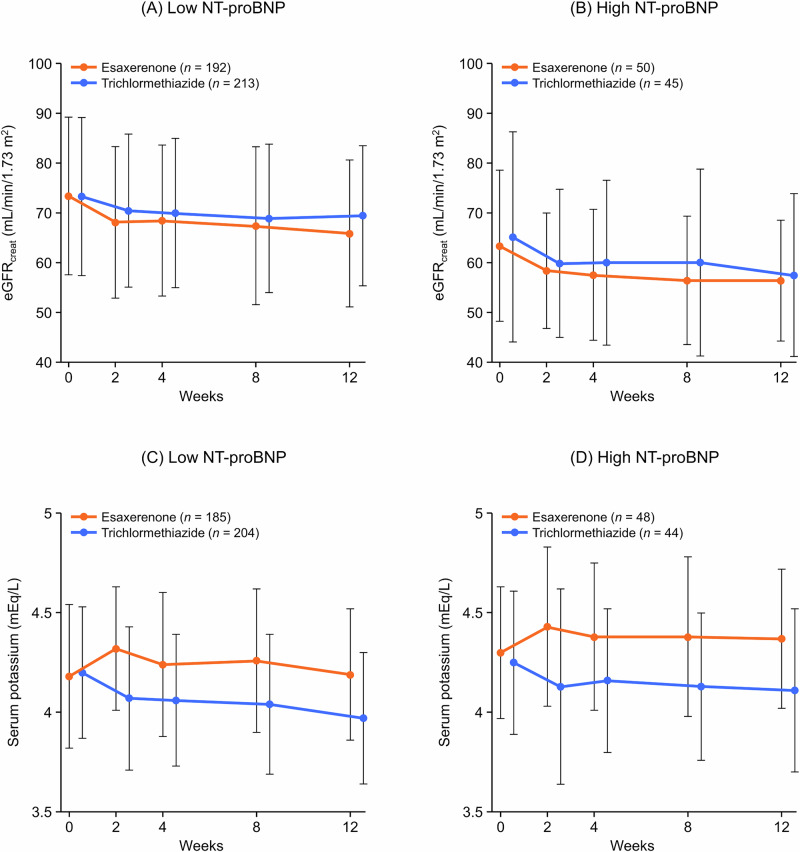
Time course changes in eGFR_creat_ (**A**, **B**) and serum potassium levels (**C**, **D**) during the study period (safety analysis set). **A**, **C** Low NT-proBNP subgroup (<125 pg/mL); **B**, **D** high NT-proBNP subgroup (≥125 pg/mL). Data are mean ± SD. In the figure key, *n* = number of patients at baseline. The number of patients differed at each measurement timepoint. eGFR_creat_ creatinine-based estimated glomerular filtration rate, NT-proBNP N-terminal pro B-type natriuretic peptide, SD standard deviation

Time course changes in eGFR_creat_ and serum potassium in the NT-proBNP subgroups are shown in Fig. 3A–D. In both NT-proBNP subgroups, the eGFR_creat_ decreased over the first 2 weeks and remained relatively stable until Week 12 (Fig. 3A, B, and Supplementary Table 3). In the low NT-proBNP subgroup, the changes in eGFR_creat_ from baseline to Week 12 were −7.41 ± 8.70 (mean ± standard deviation) and −4.23 ± 9.25 mL/min/1.73 m^2^ in the esaxerenone and trichlormethiazide groups, respectively; in the high NT-proBNP subgroup, they were −7.89 ± 9.61 and −7.28 ± 14.40 mL/min/1.73 m^2^ in each treatment group, respectively.

In both NT-proBNP subgroups, serum potassium levels increased over the first 2 weeks and remained stable until Week 12 after starting esaxerenone treatment (Fig. 3C, D and Supplementary Table 3). Conversely, patients who received trichlormethiazide had an initial decrease in serum potassium up to the 2-week timepoint, after which the levels stabilized and were maintained up to 12 weeks. In the low NT-proBNP subgroup, the respective proportions of patients with serum potassium levels <3.5 mEq/L and ≥5.5 mEq/L were 2.7% and 0.5% in the esaxerenone group and 12.1% and 0.0% in the trichlormethiazide group; in the high NT-proBNP subgroup, these proportions were 2.0% and 4.1% in the esaxerenone group and 13.6% and 2.3% in the trichlormethiazide group (Supplementary Table 4). No cases of serum potassium level ≥6.0 mEq/L were reported.

In the low NT-proBNP subgroup, the proportions of patients with UA level >7.0 mg/dL were slightly higher in the trichlormethiazide group compared with the esaxerenone group (35.6% and 28.1%, respectively); in the high NT-proBNP subgroup, the proportions were similar between the trichlormethiazide and esaxerenone groups (31.1% and 30.0%, respectively; Supplementary Table 5).

## Discussion

In summary, patients in the high NT-proBNP subgroup were older, had lower eGFR_creat_, and had a higher prevalence of HF than those with lower NT-proBNP levels. Both esaxerenone and trichlormethiazide reduced morning home BP significantly in both subgroups, with a larger BP reduction generally seen in the high NT-proBNP subgroup. In the low NT-proBNP subgroup, esaxerenone showed superiority in lowering SBP and non-inferiority in lowering DBP compared with trichlormethiazide. Similar trends were observed in bedtime home BP and office BP. Although the non-inferiority of esaxerenone to trichlormethiazide was not confirmed in the high NT-proBNP subgroup, this may be due to a smaller sample size rather than a diminished effect. Of note, the LS mean changes in morning home BP from baseline in the high NT-proBNP subgroup were similar to those observed in the primary study [12].

The greater antihypertensive effect in the high NT-proBNP subgroup likely reflects higher baseline BP and more pronounced cardiac and renal load, potentially with greater renin–angiotensin–aldosterone system overactivation. It is possible that the higher rate of ARB use in the high NT-proBNP subgroup contributed to this finding. A previous study reported that esaxerenone has a greater antihypertensive effect when used in combination with ARBs compared with CCBs [13]. Therefore, the use of ARBs in patients with high NT-proBNP levels could potentially contribute to the effects of esaxerenone. Esaxerenone’s cardiovascular and renal protective effects may be more evident in such patients, while trichlormethiazide provided a robust BP-lowering effect possibly via volume reduction or an improved peripheral vascular resistance.

In the high NT-proBNP subgroup, the wider CI for morning home BP reduction in the esaxerenone group suggests heterogeneous responses among patients. This finding indicates that certain patient characteristics may enhance or reduce the antihypertensive effect of esaxerenone. For example, patients with more pronounced volume overload, stronger activation of the renin–angiotensin–aldosterone system, or those with specific comorbidities (e.g., diabetic nephropathy or HF) might derive a greater antihypertensive benefit [9, 19, 20]. In contrast, factors such as low plasma aldosterone levels or moderate kidney impairment could attenuate the response [21, 22]. Although the present study was not specifically designed to explore the influence of the above factors, further analyses adjusting for these factors may help clarify which patient groups respond most favorably to esaxerenone.

UACR reduction was observed in both NT-proBNP subgroups, yet the magnitude was greater in the high NT-proBNP subgroup. An association between UACR and NT-proBNP has been reported previously in two clinical studies, although it should be noted that the target diseases were HF and type 2 diabetes mellitus, rather than hypertension [23, 24]. The high NT-proBNP level may reflect a significant cardiac and renal burden, potentially amplifying the effect of treatment in this subgroup. In this study, about 20% of patients with high NT-proBNP at baseline had HF. Esaxerenone elicited a significant UACR-lowering effect in this subgroup, which is comparable to the results of the ESES-LVH study [9]. Furthermore, the beneficial effects of esaxerenone on UACR were consistent regardless of NT-proBNP levels at baseline. The UACR-lowering effect of esaxerenone observed in the present subanalysis supports the underlying data on its renoprotective effect and highlights its value as a clinical option for the treatment of hypertension.

Among patients with elevated baseline NT-proBNP, esaxerenone significantly reduced NT-proBNP levels from baseline, whereas trichlormethiazide did not. This was potentially influenced by the higher baseline NT-proBNP in the esaxerenone group (mean ± standard deviation: esaxerenone group, 362.9 ± 617.8 and trichlormethiazide group, 279.5 ± 289.9 pg/mL).

Regarding safety, esaxerenone carries a known risk of hyperkalemia, while trichlormethiazide increases the risk of hypokalemia, highlighting the need for regular potassium monitoring. The risk of hyperkalemia associated with MRBs such as esaxerenone can be managed with reduced dosing and regular serum potassium monitoring according to the package insert [17, 25]. In the primary analysis of the EXCITE-HT study, the incidence of hyperkalemia (serum potassium level ≥5.5 mEq/L) was 2.0% [12], which is relatively low compared with that reported in previous clinical trials of esaxerenone [25], and no cases of serum potassium level ≥6.0 mEq/L were reported. In contrast, hypokalemia (serum potassium <3.5 mEq/L) was more common in patients receiving trichlormethiazide. In the esaxerenone group, the incidences of serum potassium level ≥5.5 mEq/L and <3.5 mEq/L were similar, irrespective of NT-proBNP levels at baseline. Similar tendencies were observed in the trichlormethiazide group. As HF progresses, the electrolyte balance in the body may change, and the risk of low serum potassium is known to be increased in these patients [26–28]. However, in the present study, no differences were found in the frequency of low serum potassium between the NT-proBNP subgroups in either of the treatment groups. The incidence of serum potassium level ≥5.5 mEq/L was higher with esaxerenone vs trichlormethiazide, and the incidence of serum potassium <3.5 mEq/L was higher with trichlormethiazide vs esaxerenone, regardless of baseline NT-proBNP level, indicating that both drugs require regular monitoring of serum potassium and management according to the patient’s condition. Even with the addition of esaxerenone to ARB therapy, the risk of elevated potassium levels in patients at risk of hyperkalemia can be managed with reduced dosing and regular serum potassium monitoring [17, 25].

The EXCITE-HT study demonstrated the efficacy and safety of esaxerenone as a second-line treatment in patients with uncontrolled essential hypertension receiving an ARB or CCB, showing non-inferiority to trichlormethiazide [12]. To the best of our knowledge, there are no previous studies reporting differences in the effectiveness and safety of an antihypertensive drug by baseline NT-proBNP level when an MRB is concomitantly administered; this study is the first report of its kind. It is important to note that esaxerenone showed a favorable BP-lowering effect regardless of baseline NT-proBNP level, suggesting its potential utility across a wide range of cardiac risk profiles. In patients with NT-proBNP <125 pg/mL, esaxerenone showed superiority in lowering morning home SBP compared with trichlormethiazide. This finding may be clinically important given that elevated morning BP is associated with an increased risk of cardiovascular events [29]. Moreover, the drug’s manageable safety profile in these populations underlines its practicality in daily clinical settings, including patients with HF and other comorbidities.

The limitations of the present subanalysis are generally consistent with those of the primary EXCITE-HT study [11, 12]. Basal antihypertensive agents were administered according to the treating physician’s choice without additional adjustments for baseline characteristics or BP. Information on the definition or type of HF as a complication was not collected. A comparison of esaxerenone’s antihypertensive effect between ARB and CCB subgroups could not be made because of the small sample size. In addition to differences in eGFR_creat_ between NT-proBNP subgroups, although patients with secondary hypertension were excluded, the possibility of including a certain number of patients with primary aldosteronism cannot be ruled out because a definitive diagnosis of primary aldosteronism was not required. Finally, the non-inferiority margins were determined based on the number of cases in the primary analysis, which may have limited their applicability in this subgroup analysis, which had a smaller number of cases. Future studies with larger sample sizes are warranted, especially for patients with high baseline levels of NT-proBNP.

## Conclusion

In the context of the EXCITE-HT study, this subanalysis provides valuable insights, suggesting that esaxerenone is efficacious across a wide range of baseline NT-proBNP levels. Given the importance of morning BP control for reducing cardiovascular events, these findings have relevant clinical implications. The results also support the safety profile of esaxerenone. Further large-scale, long-term studies are needed to confirm whether such reductions correspond to improved cardiovascular outcomes.

## Supplementary information

Supplementary Information

Supplementary Materials

## Data Availability

The anonymized data underlying the results presented in this manuscript may be made available to researchers upon submission of a reasonable request to the corresponding author. The decision to disclose the data will be made by the corresponding author and the funder, Daiichi Sankyo Co., Ltd. Data disclosure can be requested for 36 months from article publication.
