# Supplementary material for: Effect of N-terminal pro B-type natriuretic peptide levels on the efficacy and safety of esaxerenone vs trichlormethiazide for the treatment of Japanese patients with uncontrolled essential hypertension: a subanalysis of the EXCITE-HT study
Source: Hypertens Res. 2025 Nov 7;49(2):431–43. doi: 10.1038/s41440-025-02412-8 (PMC12823387; doi:10.1038/s41440-025-02412-8)
Supplement: Supplementary file 1 — Supplementary Information [file 41440_2025_2412_MOESM1_ESM.docx]

**Supplementary** **Table 1.** Change from baseline in BP in the NT-proBNP subgroups (full analysis set)

| **BP** | **Low NT-proBNP**  **(<125 pg/mL)** | | | | | | **High NT-proBNP**  **(≥125 pg/mL)** | | | | | |
| --- | --- | --- | --- | --- | --- | --- | --- | --- | --- | --- | --- | --- |
|  | **Esaxerenone** | | | **Trichlormethiazide** | | | **Esaxerenone** | | | **Trichlormethiazide** | | |
|  | ***n*** | **SBP, mmHg** | **DBP, mmHg** | ***n*** | **SBP, mmHg** | **DBP, mmHg** | ***n*** | **SBP, mmHg** | **DBP, mmHg** | ***n*** | **SBP, mmHg** | **DBP, mmHg** |
| Morning home BP^1^ |  |  |  |  |  |  |  |  |  |  |  |  |
| Baseline | 188 | 138.4 ± 13.4 | 86.6 ± 9.4 | 212 | 137.9 ± 13.0 | 86.9 ± 8.9 | 49 | 150.5 ± 17.9 | 87.7 ± 11.5 | 41 | 146.4 ± 13.2 | 86.8 ± 11.5 |
| Week 12 | 184 | 127.2 ± 11.8 | 80.8 ± 9.3 | 208 | 129.2 ± 11.8 | 81.7 ± 9.1 | 46 | 135.0 ± 16.2 | 79.1 ± 10.5 | 40 | 132.5 ± 14.5 | 78.6 ± 11.0 |
| Change from baseline | 184 | −11.2 ± 9.9 | −5.9 ± 5.7 | 208 | −8.7 ± 8.4 | −5.1 ± 4.9 | 46 | −15.3 ± 12.0 | −8.6 ± 5.9 | 40 | −14.0 ± 11.5 | −8.1 ± 6.3 |
| LS mean change from baseline [95% CI] |  | −11.6  [−12.7, −10.4] | −6.1  [−6.9, −5.4] |  | −9.3  [−10.4, −8.3] | −5.5  [−6.1, −4.8] |  | −14.6  [−17.7, −11.5] | −8.4  [−9.9, −6.8] |  | −14.4  [−17.8, −11.1] | −8.2  [−9.9, −6.5] |
| Difference in LS mean change from baseline [95% CI] |  | −2.2  [−3.8, −0.7] | −0.7  [−1.6, 0.3] |  | - | - |  | −0.2  [−4.8, 4.5] | −0.2  [−2.5, 2.1] |  | - | - |
| EOT | 188 | 127.3 ± 12.0 | 80.8 ± 9.2 | 212 | 129.0 ± 11.9 | 81.6 ± 9.1 | 49 | 134.9 ± 15.9 | 79.1 ± 10.3 | 41 | 132.4 ± 14.3 | 78.7 ± 10.8 |
| Change from baseline | 188 | −11.2 ± 9.9 | −5.8 ± 5.7 | 212 | −8.9 ± 8.6 | −5.2 ± 5.0 | 49 | −15.6 ± 12.2 | −8.6 ± 6.0 | 41 | −14.0 ± 11.4 | −8.1 ± 6.2 |
| LS mean change from baseline [95% CI] |  | −11.5  [−12.7, −10.4] | −6.1  [−6.8, -5.4] |  | −9.6  [−10.7, −8.5] | −5.6  [−6.2, −4.9] |  | −14.9  [−17.9, −11.9] | −8.3  [−9.8, −6.8] |  | −14.5  [−17.8, −11.2] | −8.2  [−9.8, −6.6] |
| Difference in LS mean change from baseline [95% CI] |  | −2.0  [−3.5, −0.4] | −0.6  [−1.5, 0.4] |  | - | - |  | −0.4  [−4.8, 4.1] | −0.1  [−2.3, 2.1] |  | - | - |
| Bedtime home BP |  |  |  |  |  |  |  |  |  |  |  |  |
| Baseline | 179 | 133.8 ± 13.8 | 81.7 ± 9.9 | 206 | 134.5 ± 13.4 | 82.5 ± 10.1 | 44 | 140.6 ± 22.0 | 80.3 ± 12.9 | 40 | 135.4 ± 18.3 | 78.4 ± 13.4 |
| Week 12 | 181 | 123.4 ± 12.0 | 75.9 ± 9.5 | 206 | 125.8 ± 12.9 | 77.1 ± 9.4 | 46 | 128.9 ± 18.3 | 74.6 ± 12.4 | 40 | 124.9 ± 16.9 | 71.3 ± 11.4 |
| Change from baseline | 175 | −10.6 ± 9.9*** | −6.2 ± 5.7*** | 202 | −8.8 ± 9.3*** | −5.3 ± 6.0*** | 41 | −12.7 ± 13.2*** | −6.0 ± 7.8*** | 39 | −10.6 ± 13.3*** | −6.7 ± 8.0*** |
| EOT | 185 | 123.5 ± 12.1 | 75.9 ± 9.4 | 210 | 125.6 ± 13.0 | 77.1 ± 9.3 | 49 | 128.5 ± 18.4 | 74.3 ± 12.2 | 41 | 124.8 ± 16.7 | 71.4 ± 11.3 |
| Change from baseline | 179 | −10.5 ± 9.9*** | −6.1 ± 5.7*** | 206 | −9.0 ± 9.4*** | −5.4 ± 6.1*** | 44 | −12.3 ± 13.3*** | −5.7 ± 8.0*** | 40 | −10.8 ± 13.2*** | −6.8 ± 8.0*** |
| Office BP |  |  |  |  |  |  |  |  |  |  |  |  |
| Baseline | 188 | 142.5 ± 14.7 | 83.2 ± 11.1 | 212 | 142.6 ± 14.8 | 84.5 ± 12.4 | 49 | 155.1 ± 19.4 | 82.8 ± 13.5 | 41 | 145.7 ± 17.1 | 80.5 ± 12.3 |
| Week 12 | 183 | 129.6 ± 13.9 | 77.0 ± 11.3 | 207 | 132.6 ± 14.7 | 79.4 ± 12.1 | 46 | 138.4 ± 22.0 | 76.0 ± 14.3 | 40 | 133.5 ± 15.5 | 74.9 ± 12.4 |
| Change from baseline | 183 | −13.0 ± 12.8*** | −6.3 ± 9.1*** | 207 | −9.9 ± 12.7*** | −5.1 ± 8.8*** | 46 | −17.5 ± 16.1*** | −6.8 ± 9.6*** | 40 | −12.1 ± 14.0*** | −5.5 ± 8.3*** |
| EOT | 188 | 129.5 ± 13.8 | 76.9 ± 11.3 | 212 | 132.7 ± 15.0 | 79.4 ± 12.2 | 49 | 138.3 ± 21.3 | 76.1 ± 14.1 | 41 | 133.7 ± 15.3 | 75.1 ± 12.3 |
| Change from baseline | 188 | −13.0 ± 12.9*** | −6.3 ± 9.0*** | 212 | −9.9 ± 12.6*** | −5.0 ± 8.7*** | 49 | −16.8 ± 16.0*** | −6.6 ± 9.5*** | 41 | −12.0 ± 13.8*** | −5.4 ± 8.3*** |

Data are mean ± SD.

^1^For morning home BP, *P* values by paired *t*-test were not calculated.

****P* <0.001 versus baseline, paired *t*-test.

LS mean changes were calculated for morning home BP at Week 12 and EOT.

LS mean changes and 95% CIs were calculated using the analysis of covariance model, with morning home SBP/DBP change from baseline as the objective variable; treatment group as the explanatory variable; and baseline BP, baseline antihypertensive medication, and baseline age as covariates.

*BP* blood pressure, *CI* confidence interval, *DBP* diastolic blood pressure, *EOT* end of treatment, *LS* least squares, *NT-proBNP* N-terminal pro B-type natriuretic peptide, *SBP* systolic blood pressure, *SD* standard deviation.

**Supplementary** **Table 2.** Change in UACR and NT-proBNP from baseline to Week 12 in the NT-proBNP subgroups (full analysis set)

| **Variables** | **Low NT-proBNP**  **(<125 pg/mL)** | | | | **High NT-proBNP**  **(≥125 pg/mL)** | | | |
| --- | --- | --- | --- | --- | --- | --- | --- | --- |
|  | **Esaxerenone** | | **Trichlormethiazide** | | **Esaxerenone** | | **Trichlormethiazide** | |
| **UACR, mg/gCr** | ***n*** |  | ***n*** |  | ***n*** |  | ***n*** |  |
| Baseline | 188 | 53.88 ± 120.10 | 212 | 63.57 ± 146.67 | 49 | 421.90 ± 1099.92 | 41 | 300.10 ± 1024.86 |
| Week 4 | 188 | 36.93 ± 80.65 | 212 | 35.97 ± 68.95 | 49 | 268.99 ± 967.94 | 41 | 208.17 ± 699.09 |
| Change from baseline | 188 | −16.95 ± 86.04 | 212 | −27.60 ± 101.60 | 49 | −152.91 ± 387.91 | 41 | −91.93 ± 400.57 |
| Percentage change in geometric mean from baseline [95% CI] |  | −31.6  [−38.7, −23.7]*** |  | −30.5  [−36.6, −23.8]*** |  | −41.6  [−53.3, −26.9]*** |  | −34.4  [−50.6, −12.8]** |
| Week 8 | 185 | 33.17 ± 82.49 | 209 | 30.26 ± 55.95 | 47 | 120.54 ± 302.59 | 40 | 182.67 ± 602.66 |
| Change from baseline | 185 | −16.84 ± 64.25 | 209 | −33.72 ± 120.06 | 47 | −129.32 ± 323.37 | 40 | −124.81 ± 497.63 |
| Percentage change in geometric mean from baseline [95% CI] |  | −35.1  [−42.3, −26.9]*** |  | −35.1  [−41.1, −28.5]*** |  | −50.7  [−61.4, −37.1]*** |  | −38.0  [−54.3, −15.9]** |
| Week 12 | 183 | 35.49 ± 144.01 | 207 | 28.71 ± 53.25 | 46 | 134.44 ± 466.10 | 40 | 161.32 ± 570.85 |
| Change from baseline | 183 | −8.60 ± 146.73 | 207 | −35.48 ± 122.73 | 46 | −120.58 ± 304.66 | 40 | −146.17 ± 504.78 |
| Percentage change in geometric mean from baseline [95% CI] |  | −35.9  [−44.0, −26.7]*** |  | −40.8  [−46.8, −34.2]*** |  | −53.3  [−65.4, −37.2]*** |  | −50.2  [−60.1, −37.8]*** |
| **NT-proBNP, pg/mL** | ***n*** |  | ***n*** |  | ***n*** |  | ***n*** |  |
| Baseline | 188 | 43.44 ± 31.77 | 212 | 46.01 ± 33.76 | 49 | 362.94 ± 617.81 | 41 | 279.46 ± 289.85 |
| Week 12 | 176 | 42.82 ± 39.76 | 200 | 45.01 ± 41.08 | 44 | 216.25 ± 207.32 | 38 | 233.97 ± 230.86 |
| Change from baseline | 176 | −0.46 ± 28.77 | 200 | −1.18 ± 32.64 | 44 | −163.61 ± 526.82* | 38 | −53.45 ± 212.96 |

Data are geometric mean ± standard deviation for UACR and mean ± standard deviation for NT-proBNP.

**P* <0.05, ***P* <0.01, ****P* <0.001 versus baseline, paired *t*-test.

For UACR, *P* values are only presented for percentage change in geometric mean from baseline.

*CI* confidence interval, *NT-proBNP* N-terminal pro B-type natriuretic peptide, *UACR* urinary albumin-to-creatinine ratio.

**Supplementary** **Table 3.** Change in serum potassium and eGFR_creat_ from baseline to Week 12 in the NT-proBNP subgroups (safety analysis set)

|  | **Low NT-proBNP**  **(<125 pg/mL)** | | | | **High NT-proBNP**  **(≥125 pg/mL)** | | | |
| --- | --- | --- | --- | --- | --- | --- | --- | --- |
|  | **Esaxerenone *n* = 192** | | **Trichlormethiazide *n* = 216** | | **Esaxerenone *n* = 50** | | **Trichlormethiazide  *n* = 45** | |
|  | ***n*** | **Mean ± SD** | ***n*** | **Mean ± SD** | ***n*** | **Mean ± SD** | ***n*** | **Mean ± SD** |
| **Serum potassium (mEq/L)** |  |  |  |  |  |  |  |  |
| Baseline | 185 | 4.18 ± 0.36 | 204 | 4.20 ± 0.33 | 48 | 4.30 ± 0.33 | 44 | 4.25 ± 0.36 |
| Week 2 | 178 | 4.32 ± 0.31 | 203 | 4.07 ± 0.36 | 48 | 4.43 ± 0.40 | 42 | 4.13 ± 0.49 |
| Change from baseline | 178 | 0.14 ± 0.34 | 202 | −0.14 ± 0.31 | 47 | 0.14 ± 0.32 | 42 | −0.13 ± 0.34 |
| Week 4 | 181 | 4.24 ± 0.36 | 203 | 4.06 ± 0.33 | 47 | 4.38 ± 0.37 | 40 | 4.16 ± 0.36 |
| Change from baseline | 180 | 0.05 ± 0.36 | 202 | −0.14 ± 0.31 | 46 | 0.10 ± 0.37 | 40 | −0.10 ± 0.33 |
| Week 8 | 178 | 4.26 ± 0.36 | 200 | 4.04 ± 0.35 | 46 | 4.38 ± 0.40 | 39 | 4.13 ± 0.37 |
| Change from baseline | 178 | 0.09 ± 0.38 | 199 | −0.16 ± 0.34 | 45 | 0.10 ± 0.40 | 39 | −0.14 ± 0.36 |
| Week 12 | 176 | 4.19 ± 0.33 | 196 | 3.97 ± 0.33 | 45 | 4.37 ± 0.35 | 39 | 4.11 ± 0.41 |
| Change from baseline | 176 | 0.02 ± 0.35 | 196 | −0.23 ± 0.33 | 44 | 0.11 ± 0.34 | 39 | −0.16 ± 0.38 |
| **eGFR_creat_ (mL/min/1.73 m^2^)** |  |  |  |  |  |  |  |  |
| Baseline | 192 | 73.42 ± 15.83 | 213 | 73.33 ± 15.82 | 50 | 63.37 ± 15.14 | 45 | 65.14 ± 21.06 |
| Week 2 | 187 | 68.19 ± 15.21 | 215 | 70.46 ± 15.36 | 49 | 58.39 ± 11.57 | 43 | 59.82 ± 14.89 |
| Change from baseline | 187 | −5.18 ± 8.99 | 212 | −2.94 ± 8.56 | 49 | −5.39 ± 7.17 | 43 | −5.11 ± 11.62 |
| Week 4 | 188 | 68.50 ± 15.16 | 214 | 70.03 ± 14.98 | 49 | 57.51 ± 13.16 | 41 | 60.00 ± 16.56 |
| Change from baseline | 188 | −4.87 ± 8.55 | 211 | −3.53 ± 8.28 | 49 | −6.27 ± 9.21 | 41 | −4.66 ± 11.86 |
| Week 8 | 185 | 67.42 ± 15.83 | 211 | 68.94 ± 14.85 | 47 | 56.44 ± 12.90 | 40 | 60.06 ± 18.78 |
| Change from baseline | 185 | −5.86 ± 9.08 | 208 | −4.66 ± 9.05 | 47 | −7.55 ± 9.69 | 40 | −4.71 ± 14.14 |
| Week 12 | 183 | 65.92 ± 14.75 | 207 | 69.50 ± 14.05 | 46 | 56.37 ± 12.12 | 40 | 57.49 ± 16.33 |
| Change from baseline | 183 | −7.41 ± 8.70 | 204 | −4.23 ± 9.25 | 46 | −7.89 ± 9.61 | 40 | −7.28 ± 14.40 |

*P* values were not calculated.

*eGFR_creat_* creatinine-based estimated glomerular filtration rate*, NT-proBNP* N-terminal pro B-type natriuretic peptide, *SD* standard deviation.

**Supplementary** **Table 4.** Incidence of serum potassium level <3.5, ≥5.5, and ≥6.0 mEq/L in the NT-proBNP subgroups (safety analysis set)

| **Serum potassium level** | **Low NT-proBNP**  **(<125 pg/mL)** | | **High NT-proBNP**  **(≥125 pg/mL)** | |
| --- | --- | --- | --- | --- |
|  | **Esaxerenone**  ***n* = 192** | **Trichlormethiazide**  ***n* = 216** | **Esaxerenone**  ***n* = 50** | **Trichlormethiazide**  ***n* = 45** |
| Serum potassium <3.5 mEq/L | 5 / 186 (2.7)  [0.9, 6.2] | 25 / 206 (12.1)  [8.0, 17.4] | 1 / 49 (2.0)  [0.1, 10.9] | 6 / 44 (13.6)  [5.2, 27.4] |
| Serum potassium ≥5.5 mEq/L | 1 / 186 (0.5)  [0.0, 3.0] | 0 / 206 (0)  [0.0, 1.8] | 2 / 49 (4.1)  [0.5, 14.0] | 1 / 44 (2.3)  [0.1, 12.0] |
| Serum potassium ≥6.0 mEq/L | 0 / 186 (0)  [0.0, 2.0] | 0 / 206 (0)  [0.0, 1.8] | 0 / 49 (0)  [0.0, 7.3] | 0 / 44 (0)  [0.0, 8.0] |

Data are *n / N* (%) [95% CI].

*CI* confidence interval, *NT-proBNP* N-terminal pro B-type natriuretic peptide.

**Supplementary** **Table 5.** Incidence of UA level >7.0 mg/dL in the NT-proBNP subgroups (safety analysis set)

| **UA level** | **Low NT-proBNP**  **(<125 pg/mL)** | | **High NT-proBNP**  **(≥125 pg/mL)** | |
| --- | --- | --- | --- | --- |
|  | **Esaxerenone**  ***n* = 192** | **Trichlormethiazide**  ***n* = 216** | **Esaxerenone**  ***n* = 50** | **Trichlormethiazide**  ***n* = 45** |
| UA >7.0 mg/dL | 54 (28.1)  [21.9, 35.1] | 77 (35.6)  [29.3, 42.4] | 15 (30.0)  [17.9, 44.6] | 14 (31.1)  [18.2, 46.6] |

Data are *n* (%) [95% CI].

*CI* confidence interval, *NT-proBNP* N-terminal pro B-type natriuretic peptide, *UA* uric acid.
